# Plasma Proteomics and Metabolomics of Aromatase Inhibitors-Related Musculoskeletal Syndrome in Early Breast Cancer Patients

**DOI:** 10.3390/metabo15030153

**Published:** 2025-02-24

**Authors:** Feng Jing, Lingyun Jiang, Yuling Cao, Maoting Tian, Jiajia Qiu, Jing Zhang, Lichen Tang, Renquan Lu, Yan Hu

**Affiliations:** 1 School of Nursing, Fudan University and Fudan University Centre for Evidence-Based Nursing: A Joanna Briggs Institute Centre of Excellence, Shanghai 200032, China; 21111170001@m.fudan.edu.cn (F.J.); 22211170029@m.fudan.edu.cn (L.J.); 23211170001@m.fudan.edu.cn (Y.C.); tiana1920@163.com (M.T.); 2 Department of Nursing Administration, Shanghai Cancer Center, Fudan University, Shanghai 200032, China; rainmouse@hotmail.com; 3 Department of Clinical Laboratory, Shanghai Cancer Center, Fudan University, Shanghai 200032, China; friday_in_usst@163.com; 4 Department of Breast Surgery, Shanghai Cancer Center, Fudan University, Shanghai 200032, China

**Keywords:** breast neoplasms, aromatase inhibitors, musculoskeletal symptoms, proteomics, metabolomics, biomarkers

## Abstract

Background: Aromatase inhibitors-related musculoskeletal syndrome (AIMSS) is a common side effect experienced by early breast cancer patients undergoing endocrine therapy. This condition can result in medication discontinuation and a diminished quality of life. The objective of this study was to characterize AIMSS, investigate its pathogenesis, and identify potential biomarkers at both the protein and metabolic levels. Methods: We collected peripheral blood samples from 60 women diagnosed with breast cancer undergoing aromatase inhibitor therapy, of whom 30 had AIMSS and 30 did not. The samples were analyzed using four-dimensional data-independent acquisition (DIA)-based proteomics and untargeted metabolomics, employing liquid chromatography–mass spectrometry (LC–MS) on the latest platform. Results: The mean age of participants was 49.2 (11.3) years in the AIMSS group and 50.1 (11.5) years in the non-AIMSS group. There were no statistically significant differences between the two groups in terms of age, BMI, education level, clinical stage, and treatment. In total, we identified 3473 proteins and 1247 metabolites in the samples. The chemokine signaling pathway (*p* = 0.015), cytokine–cytokine receptor interaction (*p* = 0.015), complement and coagulation cascades (*p* = 0.004), neuroactive ligand–receptor interaction (*p* = 0.004), and the estrogen signaling pathway (*p* = 0.004) were significant enriched in differentially expressed proteins (DEPs). GnRH secretion (*p* < 0.001), sphingolipid signaling pathways (*p* < 0.001), endocrine resistance (*p* < 0.001), the estrogen signaling pathway (*p* = 0.001), endocrine and other factor-regulated calcium reabsorption (*p* = 0.001), dopaminergic synapse (*p* = 0.003), regulation of lipolysis in adipocytes (*p* = 0.004), biosynthesis of cofactors (*p* = 0.004), thyroid hormone synthesis (*p* = 0.008), aldosterone synthesis and secretion (*p* = 0.001), taurine and hypotaurine metabolism (*p* = 0.011), ovarian steroidogenesis (*p* = 0.011), and the cAMP signaling pathway (*p* = 0.011) were significantly enriched in differentially expressed metabolites (DEMs). Complement C3 (*p* = 0.004), platelet factor 4 (*p* = 0.015), KRT10 (*p* = 0.004), KRT14 (*p* = 0.004), beta-estradiol (*p* = 0.019), testosterone (*p* = 0.023), sphingosine (*p* < 0.001), and 1-stearoyl-2-arachidonoyl-sn-glycerol (*p* = 0.039) could be the monitoring and therapeutic targets for AIMSS. Conclusions: This study offered new insights into the mechanisms underlying musculoskeletal symptoms associated with aromatase inhibitors. It also highlighted potential biomarkers for predicting and addressing these symptoms in breast cancer patients, paving the way for improved intervention strategies.

## 1. Introduction

Breast cancer remains one of the most prevalent malignancies affecting women worldwide [1], with early-stage disease representing a critical window for interventions that enhance survival and quality of life. Aromatase inhibitors (AIs) have become a cornerstone of endocrine therapy for postmenopausal women with hormone receptor-positive breast cancer, offering greater efficacy in reducing recurrence and mortality compared to tamoxifen. Meta-analyses of randomized trials demonstrated that AIs significantly lower recurrence rates compared to tamoxifen, particularly during the initial treatment periods of 0–1 years (RR = 0.64, 95% CI: 0.52–0.78) and 2–4 years (RR = 0.80, 95% CI: 0.68–0.93). Additionally, 10-year breast cancer mortality was lower with AIs than with tamoxifen (12.1% vs. 14.2%; RR = 0.85, 95% CI: 0.75–0.96) [2]. These findings have informed clinical guidelines, such as the updated recommendations from the American Society of Clinical Oncology, which suggested that women with node-positive, hormone receptor-positive breast cancer consider extended endocrine therapy, including AIs, for up to 10 years [3]. However, prolonged AIs use is associated with an increased risk of bone-related toxic effects, highlighting the need for careful monitoring and management in extended treatment plans.

Aromatase inhibitor use is frequently associated with musculoskeletal symptoms, including arthralgia, myalgia, joint stiffness, tenosynovitis, and carpal tunnel syndrome. These symptoms commonly affect the hands, wrists, knees, ankles, and shoulders but may also involve the spine and pelvis. Collectively, these conditions were referred to as aromatase inhibitor-related musculoskeletal syndrome (AIMSS) [4,5,6]. The origins of AIMSS were multifactorial, involving a combination of socio-demographic and psychological factors, clinical characteristics, estrogen deprivation, genetic predispositions, and disruptions in immune and inflammatory pathways [6,7,8,9]. The prevalence of AIMSS varied significantly across studies but was consistently identified as a major clinical concern. A systematic review and meta-analysis reported prevalence rates of aromatase inhibitor-induced arthralgia ranging from 20% to 73.7% and a pooled prevalence estimate of 45.9% (95% CI: 0.397–0.520) [10]. Despite its significant impact on patient adherence to medication and overall well-being, the pathophysiology of AIMSS remained poorly understood, and effective management strategies were still limited [11].

Omics profiling provided a comprehensive method to identify molecular signatures associated with AIMSS. This approach had the potential to uncover novel biomarkers and pathogenic mechanisms, paving the way for targeted interventions. Previous research has explored genome-wide associations and functional genomics related to musculoskeletal adverse events in women undergoing AIs therapy, as well as the genetic factors linked to AIs discontinuation due to musculoskeletal symptoms [12,13]. One study investigated inflammatory metabolomic changes involved in the development of aromatase inhibitor-induced arthralgia, shedding light on potential biochemical pathways contributing to this condition [14]. Additionally, some researchers have examined specific pathways involved in aromatase inhibitor-induced pain, including those mediated by TRPA1 [15,16,17] and TRPV4 [18] channels. Despite significant efforts to study AIMSS, few investigations have utilized an integrated approach combining proteomics and metabolomics to identify key biomarkers and elucidate the molecular mechanisms in breast cancer patients experiencing AIMSS.

Proteomics and metabolomics are branches of omics science that analyze protein and metabolic profiles within specific biological systems. These approaches focus on disease phenotypes and are widely used to identify novel biomarkers and explore previously unclear molecular mechanisms [19,20]. This study aimed to analyze plasma proteomic and metabolomic profiles in early breast cancer patients undergoing AIs therapy to investigate AIMSS. By integrating proteomic and metabolomic data, we sought to identify distinct molecular signatures and dysregulated pathways associated with AIMSS, as well as potential therapeutic targets for symptom management. Our findings will enhance understanding of the complex pathophysiology of AIMSS and support the development of personalized treatment strategies to improve patient outcomes and quality of life.

## 2. Methods

### 2.1. Subjects and Study Design

This study was approved by the Ethics Committee of the Shanghai Cancer Center, Fudan University (No. 2304273-17). The participants were recruited from the Comprehensive Breast Cancer Treatment Center in Shanghai Cancer Center, Fudan University from May 2023 to January 2024. The blood samples were collected at the clinical laboratory. The inclusion criteria were as follows: women (1) aged 18 years or older, (2) diagnosed with early-stage, hormone receptor-positive breast cancer, (3) currently undergoing AIs therapy, and (4) able to understand and voluntarily consent to participate. Patients with a history of musculoskeletal diseases, recurrent or metastatic cancer, or severe organic illnesses were excluded. AIMSS was assessed using the National Cancer Institute Common Terminology Criteria for Adverse Events (NCI-CTCAE) Version 5. Trained research nurses asked patients the standardized questions in the outpatient survey: “What musculoskeletal symptoms (e.g., joint pain, muscle pain, bone pain, arthritis, diminished joint function, or other musculoskeletal problems) have you experienced since starting AIs therapy?” Case and control groups were matched based on age, duration of AIs therapy, and type of AIs therapy. Sociodemographic and clinical data, including age, BMI, education level, time since the last menstrual period, clinical stage, prior treatments, duration and type of AIs therapy, and bisphosphonate use, were collected using a standardized form. We calculated the sample size based on an empirical approach. For clinical samples, it is usual to require no less than 30 biological replicates, so we determined 30 samples each for the experimental and control groups.

### 2.2. Sample Collection and Proteomic and Metabolomic Analysis

**Figure 1:**
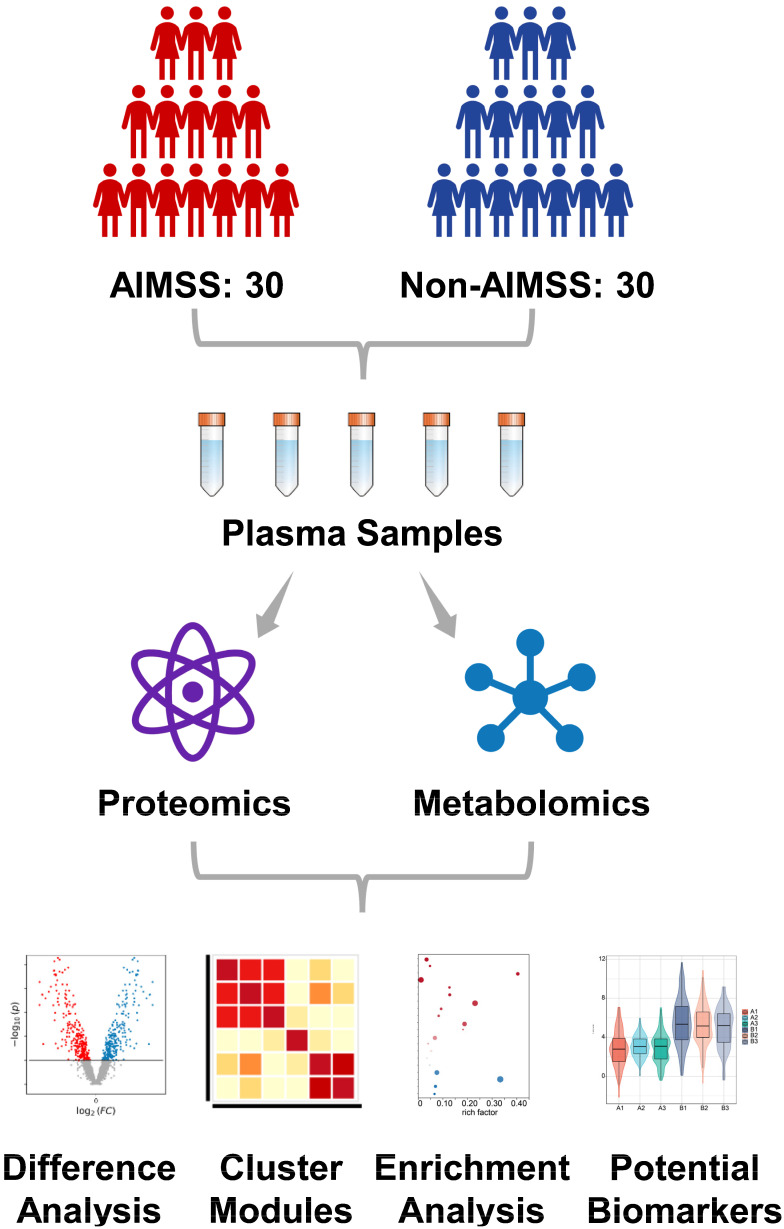
The flowchart of the plasma proteomics and metabolomics study design.

We collected 60 plasma samples from 30 AI-therapy patients with AIMSS (case group) and 30 AI-therapy patients without AIMSS (control group). Venous blood samples were collected in 5 mL vacutainer tubes containing the chelating agent ethylene diamine tetraacetic acid (EDTA), and then the samples were centrifuged for 15 min (1500 g, 4 °C). Each plasma sample was divided into two fractions: one for proteomic analysis and the other for metabolomic analysis. Samples were stored at −80 °C until experimentation. All samples were analyzed in random order, with the operators and statistical analysts blinded to group assignments. Protein identification was performed using four-dimensional data-independent acquisition (DIA) quantitative proteomic analysis, while metabolite identification utilized liquid chromatography–mass spectrometry (LC–MS) analysis. Detailed experimental methods were provided in the Supplementary Materials (see File S1). The study design flowchart is presented in Figure 1.

### 2.3. Statistical Analysis

Descriptive statistics, including mean, standard deviation (SD), counts, and percentiles, were used to summarize participant characteristics. Baseline variables between the case and control groups were compared using t-tests, chi-square tests, or Fisher’s exact tests, as appropriate.

Identified proteins were visualized using the bar graph and Venn diagram. A 3D Principal Component Analysis (PCA) was conducted to illustrate variations within and between sample groups. Quantitative signals of identified proteins in each sample were displayed in a heatmap. Differentially expressed proteins were defined as those with a *p*-value < 0.05 and a fold change (FC) > 1.5 or <0.67 [21]. To predict protein subcellular localization, we used CELLO, a multi-class SVM classification system (http://cello.life.nctu.edu.tw/) (accessed on 10 April 2024). Protein sequences were analyzed with InterProScan software (version number: interproscan-5.25-64.0) to identify protein domain signatures from the InterPro member database Pfam. The sequences of differentially expressed proteins were then searched locally using NCBI BLAST+ (ncbi-blast-2.2.28+-win32.exe) and InterProScan to find homologous sequences. Gene ontology (GO) terms were assigned, and sequences were annotated using Blast2GO. The GO annotation results were visualized using R scripts. Following annotation, the proteins were blasted against the Kyoto Encyclopedia of Genes and Genomes (KEGG) database (http://geneontology.org/) (accessed on 10 April 2024) to obtain KEGG orthology identifications and were subsequently mapped to relevant pathways. Enrichment analysis was performed using Fisher’s exact test, with Benjamini–Hochberg correction applied to adjust *p*-values for multiple testing. Only functional categories and pathways with *p*-values less than 0.05 were considered statistically significant.

All identified metabolites were classified based on their chemical taxonomy. Orthogonal partial least squares discriminant analysis (OPLS-DA) was conducted using the R package Ropls. The Variable Importance for the Projection (VIP) scores were calculated, and score plots were generated to visualize differences in metabolite composition across samples. Differential metabolites were displayed in a volcano plot, with statistical significance defined by a *p*-value < 0.05 and a VIP value > 1 [22]. Next, the pheatmap package in R (V1.0.12) was used to cluster differential metabolite abundance values and generate a heatmap. Functional analysis of the differential metabolites was carried out, with KEGG enrichment analysis performed using clusterProfiler (V4.6.0) to identify significantly enriched metabolic pathways (*p* < 0.05).

The differentially expressed metabolites and proteins were mapped to the KEGG pathway database to identify common pathways shared by both. The metabolic pathway analysis results for the differential metabolites and associated proteins were visualized using the R pathview package. Finally, boxplots were used to display the up-regulated and down-regulated proteins and metabolites within these pathways.

## 3. Results

### 3.1. Demographic and Clinical Characteristics of Participants

The study included 30 individuals with AIMSS and 30 without AIMSS, with no significant differences in age (*p* = 0.752) or BMI (*p* = 0.333). The time since the last menstrual period (LMP) and the duration of aromatase inhibitor therapy were also comparable between the two groups (*p* = 0.531 and *p* = 0.622, respectively). Educational levels showed no significant difference (*p* = 0.368), and clinical stages of breast cancer were evenly distributed between the groups (*p* = 0.709). Prior treatment regimens, including neoadjuvant therapy, chemotherapy, radiotherapy, targeted therapy, and the type of aromatase inhibitors used, were similar across both groups, with no statistical significance (*p*-values ranging from 0.184 to 1.00). Bisphosphonate therapy was reported in a small proportion of participants, with no difference between the AIMSS and non-AIMSS groups (*p* = 1.00). Additional details on the demographic and clinical characteristics of the study participants are provided in Table 1.

### 3.2. Proteomic Results of Plasma Samples in AIMSS and Non-AIMSS Group

**Figure 2:**
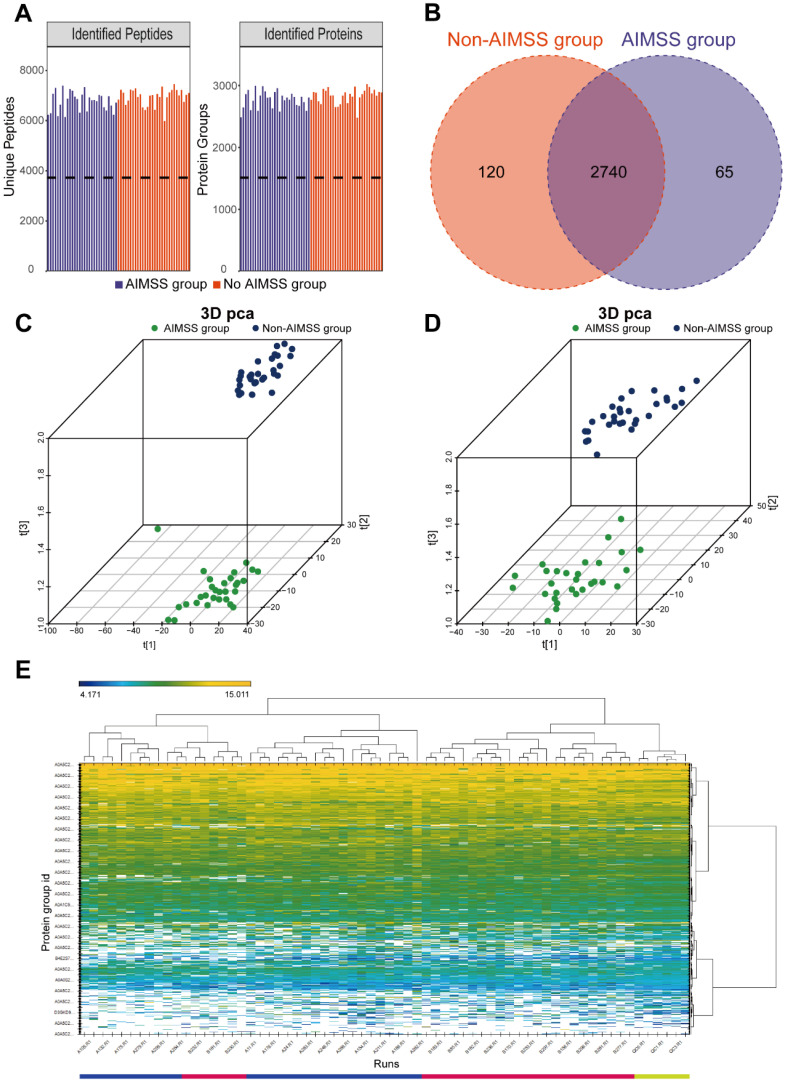
The results of quantitative analysis of the identified proteins. (**A**) The identified quantified proteins in each plasma sample. (**B**) The Venn diagram for comparison of two groups of quantitative proteins. (**C**) The 3D PCA of quantified proteins between the AIMSS group and non-AIMSS group in original data. (**D**) The 3D PCA of quantified proteins between the AIMSS group and non-AIMSS group after excluding one outlier sample. (**E**) The heatmap of the characteristics of protein expressions and distributions in each sample.

Using the 4D-DIA proteomics approach, we analyzed undepleted plasma samples from 60 participants. This analysis identified 9895 peptides and quantified 3473 proteins across all samples (Figure 2A). A Venn diagram showed 2740 proteins common to both groups, with 65 unique to the AIMSS group and 120 unique to the non-AIMSS group (Figure 2B). A 3D PCA of the quantified proteins identified one outlier in the AIMSS group (Figure 2C). After excluding this outlier, significant differences between the AIMSS and non-AIMSS groups were observed (Figure 2D). To visualize protein expression and distribution, a heatmap was generated (Figure 2E). Protein expression levels were represented using a color gradient: darker yellow indicated stronger protein signals, darker blue indicated weaker signals, and white denoted the absence of quantitative protein information.

**Figure 3:**
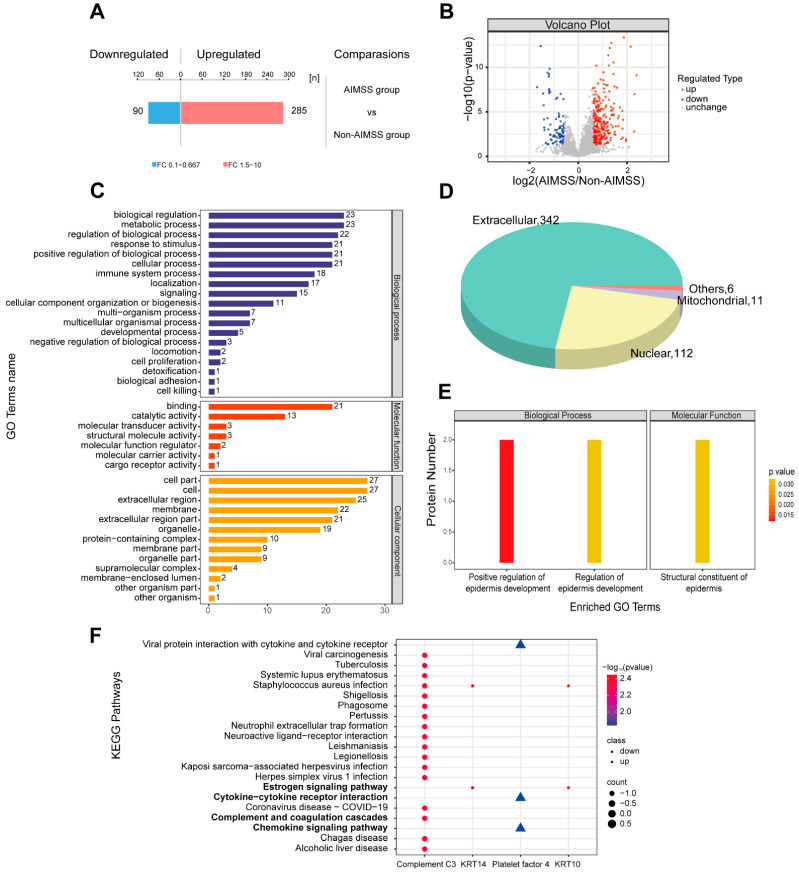
The results of quantitative analysis and function analysis of the DEPs. (**A**) Bar plot of the number of up-regulated and down-regulated proteins. (**B**) Volcano plot of significantly up-regulated and down-regulated DEPs. (**C**) GO annotation plot of DEPs. (**D**) Pie chart of subcellular localization distribution. (**E**) GO enrichment analysis of DEPs. (**F**) KEGG pathway enrichment analysis of the DEPs.

A total of 375 differentially expressed proteins (DEPs) were identified between the AIMSS and non-AIMSS groups, with 285 proteins up-regulated and 90 down-regulated in the AIMSS group compared to the non-AIMSS group (Figure 3A). The volcano plot illustrated these findings: significantly down-regulated proteins (FC < 0.67, *p* < 0.05) are highlighted in blue, significantly up-regulated proteins (FC > 1.5, *p* < 0.05) are in red, and proteins without significant differences are shown in gray (Figure 3B).

Organelles, such as mitochondria and the endoplasmic reticulum, are specialized microstructures within the cytoplasm that serve as key sites for protein functions. Subcellular localization analysis revealed that the majority of DEPs were extracellular (342, 72.6%), followed by nuclear (112, 23.8%), mitochondrial (11, 2.3%), and other locations (6, 1.3%) (Figure 3D). DEPs were also annotated using GO in the categories of Biological Process (BP), Molecular Function (MF), and Cellular Component (CC). Key roles were observed for biological regulation and metabolic processes (BP), binding (MF), and cell and cell parts (CC) in the development of AIMSS (Figure 3C). GO functional enrichment analysis identified significant enrichment in positive regulation of epidermis development, regulation of epidermis development, and structural constituents of the epidermis (*p* < 0.05) (Figure 3E). KEGG pathway enrichment analysis of DEPs revealed that the chemokine signaling pathway (*p* = 0.015), cytokine–cytokine receptor interaction (*p* = 0.015), complement and coagulation cascades (*p* = 0.004), neuroactive ligand–receptor interaction (*p* = 0.004), and the estrogen signaling pathway (*p* = 0.004) were significant enriched (Figure 3F). KRT10 and KRT14 played key roles in the estrogen signaling pathway, whereas complement C3 and platelet factor 4 influenced the chemokine signaling pathway, cytokine–cytokine receptor interaction, complement and coagulation cascades, and neuroactive ligand–receptor interaction.

### 3.3. Metabolomic Results of Plasma Samples in AIMSS and Non-AIMSS Group

**Figure 4:**
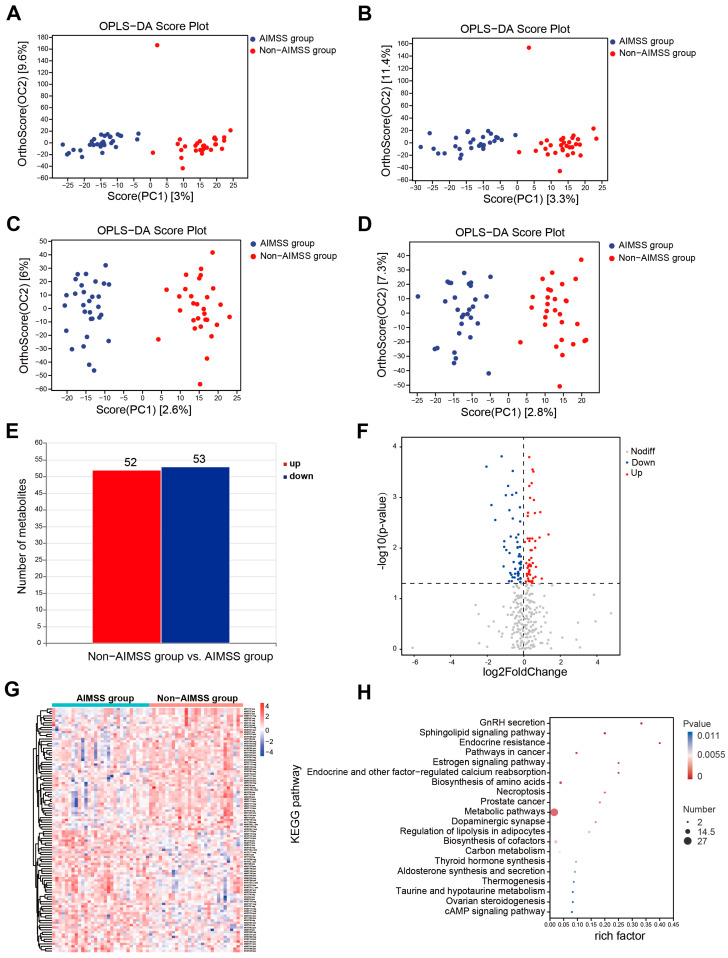
The results of differential metabolites expression analysis. (**A**) OPLS-DA analysis in original data based on positive ions. (**B**) OPLS-DA analysis in original data based on negative ions. (**C**) OPLS-DA analysis based on positive ions after excluding outlier sample. (**D**) OPLS-DA analysis based on negative ions after excluding outlier sample. (**E**) Bar plot of the number of up-regulated and down-regulated metabolites. (**F**) Volcano plot of significantly up-regulated and down-regulated DEMs. (**G**) Hierarchical clustering analysis of DEMs. (**H**) KEGG enrichment analysis of the DEMs.

We utilized the LC–MS metabolomics approach to analyze plasma samples from 60 participants, identifying 747 positive ions and 500 negative ions. The metabolites were classified based on their chemical taxonomy, with lipids and lipid-like molecules (180, 14.4%), organic acids and derivatives (110, 8.8%), and benzenoids (57, 4.6%) representing the top three metabolite classes. The OPLS-DA score plot identified one outlier sample in the non-AIMSS group in both positive and negative ion modes (Figure 4A,B). After excluding this outlier, clear separation trends between the AIMSS and non-AIMSS groups emerged in both modes (Figure 4C,D). Differential analysis of metabolites identified 52 up-regulated and 53 down-regulated differentially expressed metabolites (DEMs) in the AIMSS group compared to the non-AIMSS group, based on OPLS-DA VIP > 1 and *p* < 0.05 (Figure 4E,F). Additionally, hierarchical clustering analysis of the top 50 DEMs revealed that up-regulated metabolites were primarily classified as organic acids and derivatives, while down-regulated metabolites were predominantly lipids and lipid-like molecules (Figure 4G). Finally, KEGG enrichment analysis of DEMs highlighted the top 20 significant pathways (Figure 4H), especially for GnRH secretion (*p* < 0.001), sphingolipid signaling pathways (*p* < 0.001), endocrine resistance (*p* < 0.001), the estrogen signaling pathway (*p* = 0.001), endocrine and other factor-regulated calcium reabsorption (*p* = 0.001), dopaminergic synapse (*p* = 0.003), regulation of lipolysis in adipocytes (*p* = 0.004), biosynthesis of cofactors (*p* = 0.004), thyroid hormone synthesis (*p* = 0.008), aldosterone synthesis and secretion (*p* = 0.001), taurine and hypotaurine metabolism (*p* = 0.011), ovarian steroidogenesis (*p* = 0.011), the cAMP signaling pathway (*p* = 0.011), and so on. These metabolic pathways mainly involved 29 DEMs, such as 1-stearoyl-2-arachidonoyl-sn-glycerol, testosterone, beta-estradiol, N-(octadecanoyl) sphing-4-enine-1-phosphocholine, chorismic acid, shikimate, 2-isopropylmalic acid, sphingosine, succinate, 5.alpha.-cholest-7-en-3.beta.-ol, corticosterone, L-fucose-1-phosphate, glutathione, oxidized, DL-cysteine, 2-aminoadipic acid, guanidinoethyl sulfonate, glyceric acid, alpha-d-glucose, sepiapterin, pantothenate, porphobilinogen, orotidine, 1-methyl-l-histidine, pyocyanin, digalacturonic acid, 1-methylhydantoin, 3-methoxytyramine, 3-butynoic acid, and 5-methylbenzotriazole.

### 3.4. Integrative Analysis of Proteomics and Metabolomics

**Figure 5:**
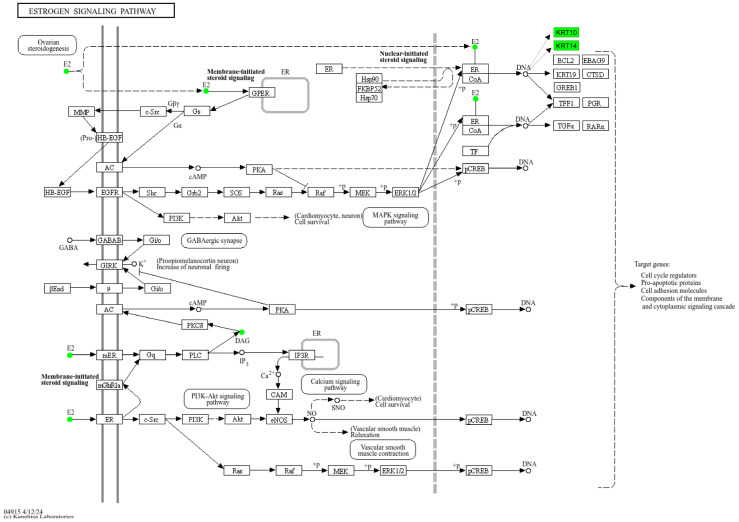
Schematic illustration of estrogen signaling pathway altered in breast cancer associated with AIMSS.

**Figure 6:**
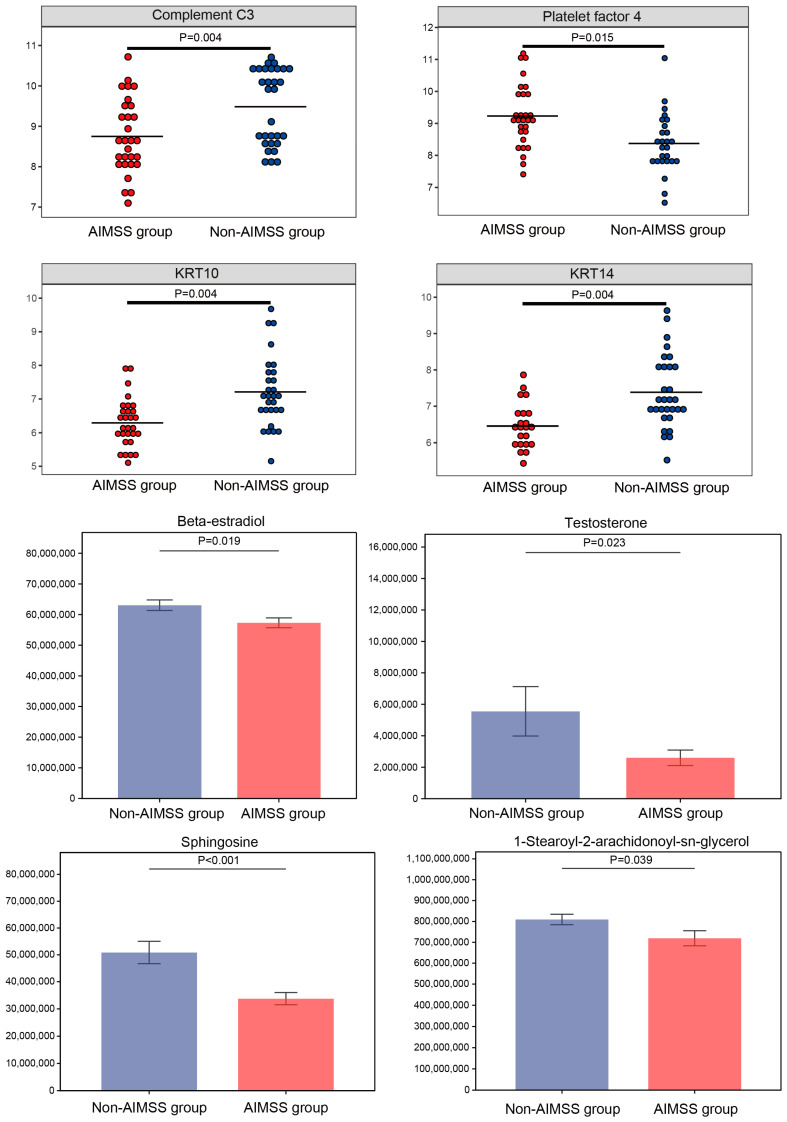
Eight selected biomarkers.

By integrating data from proteomics and metabolomics data, we found some common pathways including the estrogen signaling pathway (ko04915), tyrosine metabolism (ko00350), porphyrin metabolism (ko00860), fructose and mannose metabolism (ko00051), vitamin digestion and absorption (ko04977), glycolysis/gluconeogenesis (ko00010), and protein digestion and absorption (ko04974). For example, a modified KEGG pathway map was created to highlight the down-regulated proteins and metabolites within the estrogen signaling pathway (Figure 5). The map utilized squares to represent proteins and dots to depict metabolisms, where green indicates down-regulation. The modified map illustrated that estrogen mediated cellular actions through two main mechanisms: nuclear-initiated steroid signaling and membrane-initiated steroid signaling. In the nuclear pathway, estrogen (beta-estradiol) bound to estrogen receptors (ERs), which translocated to the nucleus, bound to estrogen response elements (EREs) on DNA, and activated the transcription of ERE-dependent genes, such as KRT10 and KRT14. In the membrane pathway, estrogen influenced cellular functions via membrane-localized estrogen receptors (mERs) or G-protein-coupled estrogen receptors (GPERs) and further influenced diacylglycerol (DAG) (i.e., 1-stearoyl-2-arachidonoyl-sn-glycerol). Upon activation of these receptors, various signaling pathways (i.e., Ca^2+^, cAMP, protein kinase cascades) were rapidly activated and ultimately influenced downstream transcription factors. Finally, we selected eight significant biomarkers from these pathways, which may be the monitoring and therapeutic targets for AIMSS (Figure 6).

## 4. Discussion

Aromatase inhibitor-related musculoskeletal syndrome (AIMSS) is a significant adverse effect of prolonged endocrine therapy in early breast cancer patients, stemming from estrogen deprivation. This condition not only diminishes patients’ quality of life but also contributes to increased rates of medication discontinuation. Despite ongoing efforts to identify risk factors and understand the pathophysiological processes underlying AIMSS, its mechanisms remain poorly defined. To address this knowledge gap, our study represents the first to employ a multi-omics approach to explore the pathogenic mechanisms of AIMSS.

Proteomics results showed that AIMSS patients have metabolic disorders, mainly involving dysregulation in cytokine–cytokine receptor interaction, complement and coagulation cascades, neuroactive ligand–receptor interaction, and the estrogen signaling pathway. Previous studies have revealed the immune-inflammatory and estrogen deprivation mechanism of AIMSS [6]. The complement cascade especially is a key component of the innate immune system that is rapidly recruited through a cascade of enzymatic reactions to enable the recognition and clearance of pathogens and promote tissue repair. It is reported that dysregulation of the complement cascade led to neuroinflammation, nociceptive sensitization, and pain [23]. A cross-sectional study also indicated that AIMSS was associated with central sensitization [24]. Additionally, keratins, as intermediate filament-forming proteins, are integral to the structural and functional integrity of epithelial cells [25]. They contribute to the formation of a proteinaceous cytoplasmic framework that helps epithelial cells resist both mechanical and non-mechanical stressors [25]. The down-regulation of KRT10 and KRT14 in AIMSS patients may indicate compromised epithelial resilience, potentially contributing to the increased sensitivity to mechanical stress or inflammatory signals seen in these patients. This finding showed the importance of keratin regulation as a target for further research in AIMSS pathophysiology and intervention strategies.

Metabolomics analysis found a novel insight that the development of AIMSS was associated with sphingolipid signaling pathways, including 1-stearoyl-2-arachidonoyl-sn-glycerol, sphingosine, and N-(octadecanoyl) sphing-4-enine-1-phosphocholine. Sphingolipids, a major component of the bilayer structure of eukaryotic cell membranes, also have powerful signal transduction functions. Sphingolipids and their various metabolites play significant roles in cell proliferation, differentiation, apoptosis, and have powerful biological activities [26]. Previous studies have shown that the molecules related to sphingolipid metabolism are involved in the specific mechanisms of various types of pain and are closely related to a variety of pain-related diseases [27]. Therefore, sphingolipid metabolism can be the focus of research on AIMSS regulation and provide new drug targets and ideas for musculoskeletal pain. Moreover, a prospective study also found that oxylipins could be biomarkers for aromatase inhibitor-induced arthralgia in breast cancer patients [14]. Therefore, it is suggested to conduct lipidomic study to deeply explore the mechanisms of AIMSS in the future.

In addition, this study identified that 1-stearoyl-2-arachidonoyl-sn-glycerol (SAG) was significantly down-regulated in the AIMSS group compared to the non-AIMSS group. SAG is a 1,2-diacyl-sn-glycerol (DAG) containing polyunsaturated fatty acids in which the acyl groups at positions 1 and 2 are specified as stearoyl and arachidonoyl, respectively [28], that participates in various signaling pathways, including the estrogen signaling pathway. Polyunsaturated fatty acids have been widely recognized for their beneficial effects on human health [29]. For example, omega-3 fatty acids, predominantly found in fish oil, play a key role in modulating inflammation and enhancing cell-mediated immunity [30]. A randomized, placebo-controlled study evaluated the effectiveness of omega-3 fatty acids supplementation for treating AIMSS in breast cancer patients [31]. The study reported a substantial and sustained improvement in arthralgia (greater than 50%) for both the omega-3 fatty acids and placebo groups. However, no meaningful difference was observed between the two groups in terms of efficacy. This suggested that while omega-3 fatty acids may have potential benefits for managing symptoms, the observed improvements could be due to a placebo effect or natural history of arthralgia rather than a direct therapeutic effect of the supplementation. Further studies with more refined methodologies or combination therapies might help clarify the role of omega-3 fatty acids in AIMSS management. Another randomized, double-blind, placebo-controlled pilot study investigated the effects of supplementation with 4 g of eicosapentaenoic acid (EPA) and docosahexaenoic acid (DHA) daily for three months in postmenopausal breast cancer survivors undergoing aromatase inhibitor therapy [32]. The study found that this regimen significantly increased levels of serum EPA, DHA, total omega-3 polyunsaturated fatty acids, and long-chain omega-3 polyunsaturated fatty acids and inhibited bone resorption compared to the placebo group, with a statistically significant difference (*p* < 0.05). However, despite these promising effects on bone metabolism, the study did not observe changes in inflammatory markers. This suggested that while omega-3 supplementation may benefit bone health in this population, its influence on inflammation-related pathways, which were hypothesized to play a role in AIMSS, remains unclear. In summary, nutritional supplementation has emerged as a promising intervention strategy for managing AIMSS. The identification of SAG as a significantly down-regulated metabolite in AIMSS underscores its potential as both a prevention and treatment target. However, given the complexity and variability in the types and compositions of polyunsaturated fatty acids, future research must focus on determining precise SAG supplementation strategies, including dosage and delivery methods, to maximize clinical benefits.

Beta-estradiol and testosterone are critical intersection points in several pathways. In postmenopausal women, estrogen is primarily synthesized in peripheral tissues, with adipose tissue being the predominant site of production [6,33]. Testosterone and androstenedione, the primary substrates, are converted into beta-estradiol and estrone, respectively, through the action of aromatase. Aromatase is a cytochrome P450 enzyme encoded by the CYP19A1 gene, which plays a crucial role in estrogen biosynthesis [6]. Aromatase inhibitors effectively reduce estrogen levels by targeting the aromatase enzyme. Steroidal AIs, such as exemestane, bind aromatase irreversibly, while nonsteroidal AIs, including anastrozole and letrozole, inhibit the enzyme reversibly. This suppression of aromatase activity leads to a significant reduction in estrogen levels and a subsequent down-regulation of ER activity [34]. Furthermore, prior genetic analyses have identified associations between specific genetic variants and the development of arthralgia during aromatase inhibitor therapy. Variants in CYP19A1 (rs4775936) and ESR1 (rs9322336, rs2234693, rs9340799) were notably linked to an increased risk of experiencing musculoskeletal symptoms, highlighting a potential genetic predisposition to these adverse effects [7]. Additionally, a case-control study demonstrated that mean estradiol levels were significantly lower in the AIMSS group compared to the non-AIMSS group (8.98 ± 5.92 pg/mL vs. 11.23 ± 6.22 pg/mL, *p* = 3.16 × 10^−7^) [35]. This finding underscored the role of estrogen depletion in the development of aromatase inhibitor-related musculoskeletal symptoms. Overall, our study, combined with prior evidence, indirectly confirmed that breast cancer patients with AIMSS exhibited heightened sensitivity to estrogen deprivation. Particularly, estradiol emerged as a promising biomarker for predicting and monitoring aromatase inhibitor-related musculoskeletal syndrome. This finding highlighted the potential for personalized approaches in managing and mitigating the adverse effects of aromatase inhibitors, thereby improving the quality of life and treatment adherence among breast cancer patients.

## 5. Limitations

This study has several limitations. First, the study was conducted at a single center with a relatively small sample size, which might limit the generalizability and robustness of the findings. Future large-scale, multicenter prospective studies are essential to validate the observed results and ensure broader applicability. Second, the study employed untargeted proteomics and metabolomics approaches without absolute quantification. While this allowed for the discovery of potential biomarkers, the findings should be further supported by targeted quantification techniques, such as targeted LC–MS/MS, to confirm and standardize the clinical applicability of identified markers like beta-estradiol, SAG, and keratins. Third, the absence of a healthy control group limited the ability to compare findings directly to general physiological levels. Including healthy controls in future studies would enhance the understanding of AIMSS pathophysiology. Additionally, due to resource constraints, subgroup analyses (e.g., based on patient demographics, clinical characteristics, and the severity of AIMSS) were not conducted. Stratified analyses could provide deeper insights into variability among patients and the specific risk factors for AIMSS development. Consequently, although this study provided an integrated proteomic and metabolomic analysis, further investigations into genomic, transcriptomic, and lipidomic profiles could offer a more complete understanding of AIMSS and reveal additional biomarkers or pathways involved.

## 6. Conclusions

In summary, this study applied an integrated proteomics and metabolomics strategy to explore the mechanisms underlying aromatase inhibitor-related musculoskeletal syndrome (AIMSS) in patients with early breast cancer. The study not only deepened the understanding of the molecular pathways involved in AIMSS but also identified novel potential biomarkers, including complement C3, platelet factor 4, KRT10, KRT14, beta-estradiol, testosterone, sphingosine, and 1-stearoyl-2-arachidonoyl-sn-glycerol. These findings provided valuable insights for the prediction and intervention of musculoskeletal symptoms in breast cancer patients, offering a foundation for future research to optimize clinical management and improve patient outcomes.

## Figures and Tables

**Table 1:** Demographic and clinical characteristics of participants.

Characteristics	AIMSS (n = 30)	Non-AIMSS (n = 30)	*p*-Value
	Mean (SD)	Mean (SD)	
Age, years	49.2 (11.3)	50.1 (11.5)	*p* = 0.752 ^a^
BMI, kg/m^2^	22.4 (3.0)	23.2 (3.3)	*p* = 0.333 ^a^
Time since last menstrual period (LMP), years	4.1 (5.2)	5.2 (7.4)	*p* = 0.531 ^a^
Time of aromatase inhibitors therapy, months	6.4 (2.4)	6.1 (2.8)	*p* = 0.622 ^a^
	n (%)	n (%)	
Education			*p* = 0.368 ^b^
Junior high school and below	2 (6.7%)	5 (16.7%)	
High school	11 (36.7%)	7 (23.3%)	
College and above	17 (56.7%)	18 (60%)	
Clinical stage			*p* = 0.709 ^b^
I	14 (46.7%)	18 (60.0%)	
II	15 (50.0%)	11 (36.7%)	
III	1 (3.3%)	1 (3.3%)	
Prior regimen			
Neoadjuvant therapy	6 (20.0%)	3 (10.0%)	*p* = 0.472 ^b^
Chemotherapy	20 (66.7%)	17 (56.7%)	*p* = 0.426 ^c^
Radiotherapy	21 (70.0%)	16 (53.3%)	*p* = 0.184 ^c^
Targeted therapy	8 (26.7%)	8 (26.7%)	*p* = 1.00 ^c^
Type of aromatase inhibitors			*p* = 0.853 ^b^
Anastrozole	5 (16.7%)	7 (23.3%)	
Letrozole	4 (13.3%)	3 (10.0%)	
Exemestane	21 (70.0%)	20 (66.7%)	
Bisphosphonate therapy			*p* = 1.00 ^c^
Yes	4 (13.3%)	5 (16.7%)	
No	26 (86.7%)	25 (83.3%)	

^a^ t-test; ^b^ Fisher’s exact test; ^c^ Chi-square test.

## Data Availability

The data presented in this study are available on request from the corresponding authors.

## Supplementary Materials

The following supporting information can be downloaded at https://www.mdpi.com/article/10.3390/metabo15030153/s1. File S1: detailed experimental methods.

## Abbreviations

AIs	aromatase inhibitors
AIMSS	aromatase inhibitors-related musculoskeletal syndrome
DIA	data-independent acquisition
LC–MS	liquid chromatography–mass spectrometry
DEPs	differentially expressed proteins
DEMs	differentially expressed metabolites
GO	Gene Ontology
KEGG	Kyoto Encyclopedia of Genes and Genomes
PCA	Principal Component Analysis
FC	fold change
OPLS-DA	orthogonal partial least squares discriminant analysis
VIP	Variable Importance for the Projection
ERs	estrogen receptors
DAG	diacylglycerol
SAG	1-stearoyl-2-arachidonoyl-sn-glycerol
EPA	eicosapentaenoic acid
DHA	docosahexaenoic acid
