# Supplementary material for: Plasma Proteomics and Metabolomics of Aromatase Inhibitors-Related Musculoskeletal Syndrome in Early Breast Cancer Patients
Source: Metabolites. 2025 Feb 24;15(3):153. doi: 10.3390/metabo15030153 (PMC11943704; doi:10.3390/metabo15030153)
Supplement: Supplementary file 1 [file metabolites-15-00153-s001.zip › metabolites-3433059-supplementary.pdf]

4D-DIA Quantitative Proteomic Analysis

Sample preparation and fractionation for DDA library generation

The 5kDa ultrafiltration tube (Sartorius) was used for desalination and concentration of high and low-abundance components. SDT buffer (4% SDS, 100 mM Tris-HCl pH 7.6) was added, boiled for 15min and centrifuged at 14,000g for 20 min. The supernatant was quantified with the BCA Protein Assay Kit (Bio-Rad, USA). The sample was stored at -80°C.

Filter-aided Sample Preparation (FASP Digestion) Procedure

DTT (with the final concentration of 40 mM) was added to each sample respectively and mixed at 600 rpm for 1.5 h (37°C). After the samples cooled to room temperature, IAA was added with the final concentration of 20 mM into the mixture to block reduced cysteine residues and the samples were incubated for 30 minutes in darkness. Next, the samples were transferred to the filters (Microcon units, 10 kDa) respectively. The filters were washed with 100 μl UA buffer three times and then 100 μl 25mM NH4HCO3 buffer twice. Finally, trypsin was added to the samples (the trypsin : protein (wt/wt) ratio was 1:50) and incubated at 37°C for 15-18 h (overnight), and the resulting peptides were collected as a filtrate. The peptides of each sample were desalted on C18 Cartridges (Empore™ SPE Cartridges C18 (standard density), bed I.D. 7 mm, volume 3 ml, Sigma), concentrated by vacuum centrifugation and reconstituted in 40 μl of 0.1% (v/v) formic acid. The peptide content was estimated by UV light spectral density at 280 nm. For DIA experiments, iRT (indexed retention time) calibration peptides were spiked into the samples.

Digested pool peptides were then fractionated to 10 fractions using Thermo Scientific™ Pierce™ High pH Reversed-Phase Peptide Fractionation Kit (for plasma sample, we just fractionated the peptides from the low-abundance components). Each fraction was desalted on C18 Cartridges (Empore™ SPE Cartridges C18 (standard density), bed I.D. 7 mm, volume 3 ml, Sigma) and reconstituted in 40μl of 0.1% (v/v) formic acid. The iRT-Kits (Biognosys) peptides were spiked before data-dependent acquisition (DDA) analysis.

Data Dependent Acquisition (DDA) Mass Spectrometry Assay

All fraction samples were analyzed by TIMSTOF mass spectrometer (Bruker) via an Evosep One system liquid chromatography (Denmark). The MS was operated in data-dependent mode for the ion mobility-enhanced spectral library generation. We set the accumulation and ramp time to 100 ms each and recorded mass spectra in the range from m/z 100–1700 in positive electrospray

mode, dynamic exclusion was 24.0s. Ion source voltage was set as 1500V, temperature was set as 180°C, and dry gas was set as 3L/min. The ion mobility was scanned from 0.75 to 1.35 Vs/cm2, then performed 8 cycles of PASEF MS/MS.

Mass Spectrometry Assay for Data Independent Acquisition (DIA)

The peptides from each sample were analysed by TIMSTOF mass spectrometer (Bruker) connected to an Evosep. One system liquid chromatography (Denmark) in the data independent acquisition (DIA) mode. The mass spectrometer collected ion mobility MS spectra over a mass range of m/z 100-1700, we defined up to 4 windows for single 100 ms TIMS scans according to the m/z ion mobility plane. During PASEF MSMS scanning, the collision energy was ramped linearly as a function of the mobility from 20 eV at $1/K_0$=0.85 [Vs/cm^2] to 59 eV at $1/K_0$=1.30 [Vs/cm^2].

Mass spectrometry data analysis

For DDA library data, the FASTA sequence database was searched with Spectronaut TM 14.4.200727.47784 (Biognosys) software. The database was downloaded at website: http://www.uniprot.org. iRT peptides sequence was added (Biognosys iRT Kit). The parameters were set as follows: enzyme is trypsin, max missed cleavages is 1, fixed modification is carbamidomethyl(C), dynamic modification is oxidation(M) and acetyl (Protein N-term). All reported data were based on 99% confidence for protein identification as determined by false discovery rate (FDR) ≤ 1%. DIA data was analyzed with Spectronaut TM 14.4.200727.47784 searching the above constructed spectral library. Main software parameters were set as follows: retention time prediction type is dynamic iRT, interference on MS2 level correction is enabled, and cross run normalization is enabled. All results were filtered based on Q value cutoff 0.01 (equivalent to FDR<1%).

Untargeted metabolomics analysis

Sample collection and preparation

The plasma samples were thawed at 4 °C and 100 µL aliquots were mixed with 400 µL of cold methanol/acetonitrile (1:1, v/v) to remove the protein. The mixture was centrifuged for 20 min (14000g, 4 °C). The supernatant was dried in a vacuum centrifuge. For LC-MS analysis, the samples were re-dissolved in 100 µL acetonitrile/water (1:1, v/v) solvent and centrifuged at 14000 g at 4 °C for 15 min, then the supernatant was injected. To monitor the stability and repeatability of instrument

analysis, quality control (QC) samples were prepared by pooling 10 μL of each sample and analyzed together with the other samples. The QC samples were inserted regularly and analyzed in every 5 samples.

LC-MS Analysis

Analysis was performed using an UHPLC (Vanquish UHPLC, Thermo) coupled to the Orbitrap. For HILIC separation, samples were analyzed using a 2.1 mm × 100 mm ACQUIY UPLC BEH Amide 1.7 μm column (waters, Ireland). In both ESI positive and negative modes, the mobile phase contained A=25 mM ammonium acetate and 25 mM ammonium hydroxide in water and B=acetonitrile. The gradient was 98% B for 1.5 min and was linearly reduced to 2% in 10.5 min, and then kept for 2 min， and then increased to 98% in 0.1 min, with a 3 min re-equilibration period employed.

The ESI source conditions were set as follows: Ion Source Gas1 (Gas1) as 60, Ion Source Gas2 (Gas2) as 60, curtain gas (CUR) as 30, source temperature: 600℃, IonSpray Voltage Floating (ISVF)±5500 V. In MS only acquisition, the instrument was set to acquire over the m/z range 80-1200 Da, the resolution was set at 60000 and the accumulation time was set at 100ms. In auto MS/MS acquisition, the instrument was set to acquire over the m/z range 70-1200 Da, the resolution was set at 30000 and the accumulation time was set at 50ms, and excluded time within 4 s.

Data processing

The raw MS data was converted to MzXML files using Proteo Wizard MSConvert before importing it into freely available XCMS software. For peak picking, the following parameters were used: centWave m/z = 10 ppm, peakwidth = c (10, 60), prefilter = c (10, 100). For peak grouping, bw = 5, mzwid = 0.025, minfrac = 0.5 were used. CAMERA (Collection Algorithms of MEtabolite pRofile Annotation) was sued for annotation of isotopes and adducts. In the extracted ion features, only the variables having more than 50% of the nonzero measurement values in at least one group were kept. Compound identification of metabolites was performed by comparing of accuracy m/z value (<10 ppm), and MS/MS spectra with an in-house database established with available authentic standards.
